# Candidate genes potentially involved in molting and body size reduction in the male of the horned gall aphid, *Schlechtendalia chinensis*

**DOI:** 10.3389/fphys.2023.1097317

**Published:** 2023-02-06

**Authors:** Hongyuan Wei, Xin Xu, Guorui Feng, Shuxia Shao, Xiaoming Chen, Zixiang Yang

**Affiliations:** Key Laboratory of Cultivation and Utilization of Resource Insects, Institute of Highland Forest Science, Chinese Academy of Forestry, National Forestry and Grassland Administration, Kunming, China

**Keywords:** *Schlechtendalia chinensis*, horned gall aphid, molt, negative growth, weighted gene co-expression network analysis (WGCNA), hub gene

## Abstract

In general, insects grow (increase in body size) through molting. To the opposite, the body size of the males of the horned gall aphid, *Schlechtendalia chinensis*, gets smaller after molting and as they age. To understand the molecular bases of this rare phenomenon, transcriptomes were generated from 1–5 days old male and the data were analyzed *via* a weighted gene co-expression network analysis (WGCNA). A total of 15 partitioned modules with different topological overlaps were obtained, and four modules were identified as highly significant for male body length (*p* < 0.05). Kyoto Encyclopedia of Genes and Genomes (KEGG) enrichment analysis suggested that a portion of genes in the four modules are likely involved in autophagy and apoptosis. In addition, a total of 40 hub genes were obtained in the four modules, and among them eight genes were highly expressed in males compared to individuals of other generations of *S. chinensis*. These eight genes were associated with autophagy and apoptosis. Our results reveal the unique negative growth phenomenon in male *S. chinensis* after molting, and also suggest that the male *S. chinensis* with no ability to feed probably decompose their own substances *via* autophagy and apoptosis to provide energy for life activities such as germ cell development.

## Introduction

All insects molt (cast off their exoskeleton) in order to grow and develop. The molting processing costs energy and nutrients. Most insects increase their body size and experience morphological development after molting. However, there are examples on the contrary. Oviparous female aphids belong to Eriosomatinae showed decreasing body size through molts while having no functional mouthparts (Lambers, 1966). This unique phenomenon in some insects were briefly reported, but the molecular basis and the biology advantage of such a phenomenon are unclear.

Aphids are important model organisms in evolutionary biology and ecology because they exhibit unique features such as complex life cycles, sexual and asexual reproduction strategies and alternation of host plants required for normal development (Stern, 2008; Eterovic and Vilcinskas, 2016). The horned gall aphid, *Schlechtendalia chinensis* (Hemiptera: Aphididae: Eriosomatinae), is an economically important insect as it induces horned gall formations, which are valuable for the Chinese medicine and chemical industry (Zhang et al., 1999; Blackman and Eastop, 2020; Chen et al., 2020). The life cycle of *S. chinensis* comprises a single sexual generation and five asexual generations as well as a host switch from the Chinese sumac in the summer and autumn to moss plants in the winter. Both males and females are live outside the galls (Zhang et al., 1999; Yang et al., 2010). It was reported that the newborn nymphs of males and females reach the sex mature by molting 4 days after birth. As instars progress through development, their body size decreases, resulting in negative growth (Zhang and Tang, 1987). All instars of both sexes lack functional mouthparts, meaning they cannot access to nutrition through feeding. This raises an interesting question as to why and how can they maintain normal life activities such as molting, roaming, mating and reproducing without food intake? And related to this, what are the energy and material resources used instead? Tackling these questions with transcriptomics is now possible since the genome of *S. chinensis* has been sequenced recently (Wei et al., 2022). The weighted gene co-expression network analysis (WGCNA) is a powerful method to identify co-expressed groups of genes from large heterogeneous messenger RNA expression data sets (Clarke et al., 2013). It has been successfully used for insect transcriptome analysis, to effectively mine genes of interest, and to predict gene functions (Ding et al., 2022).

In this study, we investigated the transcriptional changes over time during the sexual stages of *S. chinensis*. We generated transcriptomes of males aged from 1 to 5-day-old and clustered genes with similar expression patterns. Using WGCNA, we performed a correlation analysis between phenotypic traits and hub genes. The current study not only identified a likely mechanism by which aphids fuel their developmental processes without feeding, but also provides a foundation for further studies on the physiology and molecular biology of aphid development.

## Material and methods

### Insect sample collection

Spring migrants (sexuparae) of *S. chinensis* from mosses were collected in the field in Yanjin County (28°06′N, 104°22′E, 980 m elev.), Yunnan Province, China. The offspring (males and females) of the collected sexuparae were cultivated to obtain aphid samples of different ages, from 1 to 5-day-old for the study, 150 individuals in total which were immediately frozen in liquid nitrogen and stored at −80°C for further RNA analysis.

### Morphological observations and measurements

Fresh aphids were cleaned gently by a tiny brush under a stereo microscope (SZ61, Olympus, Japan), then were dehydrated with a graded ethanol series (70%, 80%, 90%, 95%, and 100%) and coated with gold in a JS-1600 ion sputter coater (Saintins, Nanjing, China), and observed and photographed under a TM3000 scanning electron microscope (Hitachi, Japan). Measurements were taken using a VHX-1000 digital microscope (Keyence, Japan). Three biological replicates (ten aphid individuals per replicate) were measured for each sample. All measurements are in micrometers (μm). Data were analyzed using SPSS 20.0. The differences among samples were examined using post-hoctest on linear mixed-effects model.

### High throughput transcriptome sequencing

Transcriptomes were generated from RNA samples extracted from male samples with three biological replicates (10 aphid individuals per replication). RNA amount, purity and integrity were determined on a NanoPhotometer N60 (Implen GmbH, Munich, Germany) and an Agilent 2,100 Bioanalyzer (Agilent Tech. CA, United States). cDNA libraries were initially quantified by a Qubit™ 2.0 Fluorometer (Thermo Fisher Scientific Inc. MA, United States) and diluted to 1.5 ng/μL. Later, different libraries were pooled according to the requirements of effective concentration and target data volume. RNA was sequenced using Illumina’s high throughput sequencing platform NovaSeq 6,000 (Illumina, Inc. CA, United States). In order to obtain putative transcripts, clean reads were mapped to *S. chinensis* genome assembly using Hisat2 (version 2.1.0.5) (Kim et al., 2015; Wei et al., 2022). The low-quality alignments were filtered with Sequence Alignment/Map tools (SAMtools) (Li et al., 2009). Transcripts per million (TPM) expression values were calculated using featureCounts (Yang et al., 2014) and StringTie (Pertea et al., 2015) for transcripts. Transcriptomes of other generations of *S. chinensis*, autumn migrant, fundatrix, fundatrigenia, overwinter nymph, female and spring migrant (sexuparae) came from the previous research (Wei et al., 2022).

### WGCNA data input and preprocessing

Three data sets, including the fragments per kilobase of exon per million mapped fragments (FPKM) value of all aphid samples, the mean body length values of each biological replicate of samples, and the gene annotation were used for WGCNA analysis (Supplementary Materials S1–S3). After FPKM values were calculated, genes which expression levels equaled to 0 were removed. The median absolute deviation (MAD) for each gene was calculated and sorted by the values. Unqualified genes were removed, and the resulting data was used for cluster analysis. The top 75% of these genes (based on a MAD value > 0.01) were used to perform a cluster analysis based on Euclidean distance of samples to construct a hierarchical clustering dendrogram. Outliers of the cluster tree were removed by manual check. We then proceeded by excluding these three samples from the data, and the data from the remaining 12 samples to construct a hierarchical clustering dendrogram based on Euclidean distance too.

### Gene network construction and module identification

All analyses were conducted using the R software package WGCNA 1.71 (Langfelder and Horvath, 2008). RStudio 4.2.1 was selected for code writing and execution. All language source codes of the calculation process are shown in Supplementary Material S4. The network topology for various soft-thresholding powers were detected using a “*pickSoftThreshold()*” function and an appropriate soft-thresholding power β was selected through the scale-free fit index in the WGCNA software package. The automatic block-wise network construction and module detection were performed using a “*blockwiseModules()*” function with β as a parameter.

The network construction procedure included the following main steps: 1) Select the appropriate soft threshold (weighting coefficient) and define the similarity matrix; 2) the similarity matrix was converted into an adjacency matrix using a power adjacency function; 3) the adjacency matrix was transform into a topological overlap matrix (TOM); 4) the hierarchical clustering tree was obtained by performing hierarchical clustering for TOM-based dissimilarity (dissTOM); 5) modules were identified as branches of the hierarchical cluster tree using the dynamic tree cut method while the module eigengene (ME) which represents the overall expression level of each module was calculated; 6) the Pearson correlation coefficients between MEs of all modules were calculated, and the 1-Pearson correlation coefficient was defined as the average distance between MEs of all modules; and 7) the modules with high similarity were merged to obtain the co-expression network.

### Identification of body length significant modules

The correlation between modules and morphologic trait data (body length) were calculated using the “*cor()*” function in R. The Student asymptotic *p*-value of the correlation was calculated and the module-trait association heatmap was generated by the “*corPvalueStudent()*” function. Finally, modules with high weight correlations (*p < 0.05*) were selected to draw a scatter plot of gene significance and module membership.

### Network visualization and hub gene selection

Modules with *p* < 0.05 from the topological overlap results were screened. The edge and node data files were analyzed using Cytoscape 3.9.1 (Shannon et al., 2003) software by “*exportNetworkToCytoscape()*” function. First, we calculated the degree of each node, then sorted node values from large to small. In this manner, we produced a list of the top-scoping 100 genes. Then, using the Cytoscape software, the 100 nodes and their corresponding edges were used to create a network diagram. CytoNCA, the Cytoscape plug-in unit 2.1.6 (Tang et al., 2015), was used to analyze the new network diagram. We then checked the “with weight” option when calculating and setting the weight parameter in the ‘Edges attributes’ panel. From the analysis results of CytoNCA, the top 10% of genes, ranking from large to small, were selected as hub genes. GO and KEGG enrichment were analyzed, and ridgeline plots were made using Omicshare CloudTools with default parameters (http://www.omicshare.com/).

## Results

### Morphological characteristics: Observations and measurements

**FIGURE 1:**
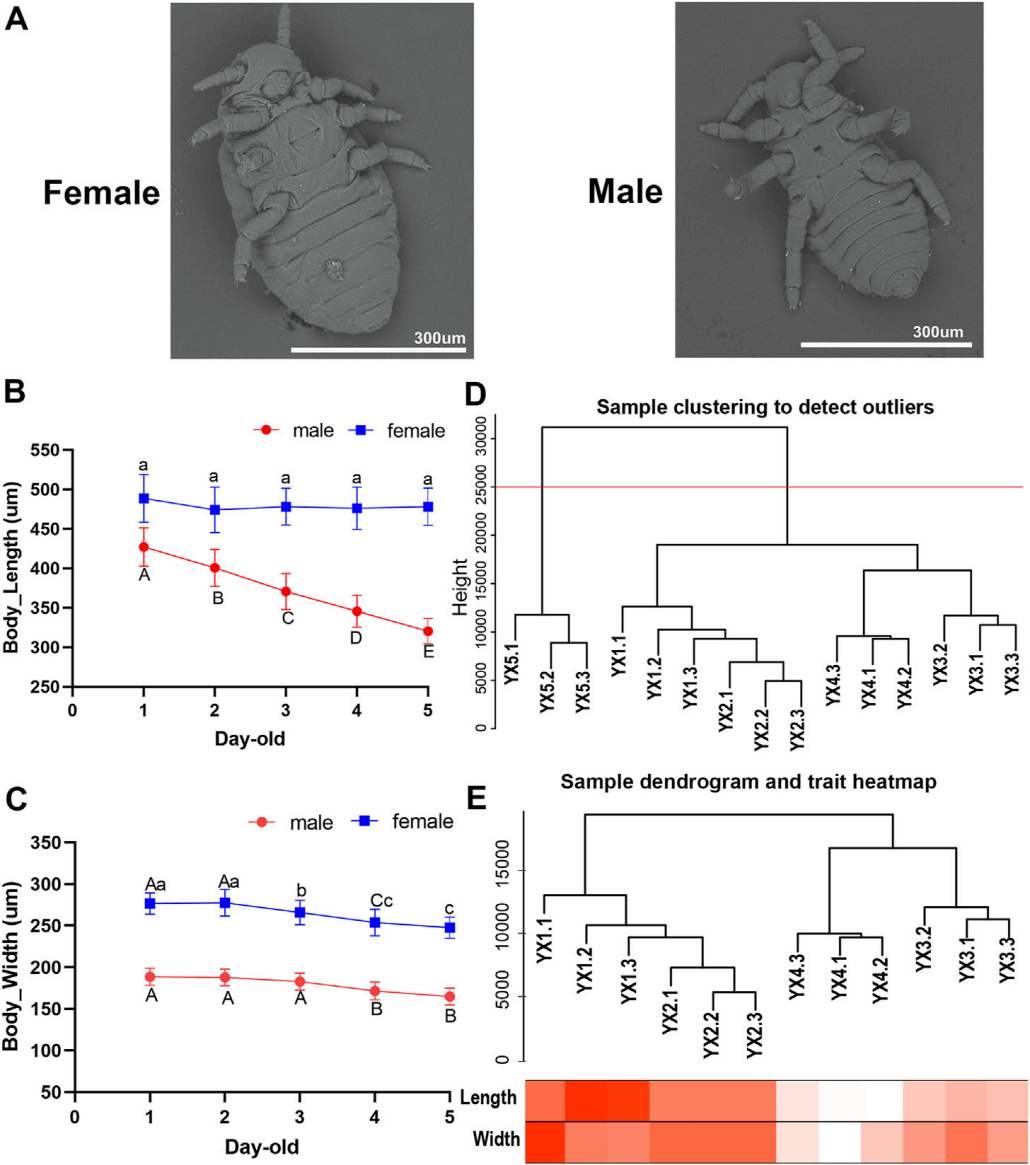
Characteristics and transcriptome analysis of male aphid, *Schlechtendalia chinensis* at different age. **(A)** Scanning electron microscope (SEM) image of females and males. **(B)** and **(C)** The body length and body width of 1–5 days old males and females (Different lower case letters and upper case letters indicate significant differences at 0.05 and 0.01 levels, respectively). **(D)** Dendrogram of samples based on their Euclidean distance. YX1, YX2, YX3, YX4, YX5 in the figure represent 1–5 days old males, respectively. The last digit of 1, 2, and 3 represents the three biological repetitions of the sample. The red horizontal division line represents the abscissa at 25,000 in the figure. **(E)** Sample dendrogram and trait heatmap of males. The darker the color, the stronger the correlation.

Utilizing SEM on both male and female *S. chinensis* specimens, we found that both lack functional mouthparts before and after molting. The body size of males is smaller than that of females (Figure 1A). With the increase of age, the body size of males and females gradually decreased, and showed negative growth through a number of molts (Supplementary Material S6). The average body lengths of 1–5 day-old males were 427.02, 400.65, 370.70, 345.59, and 320.25 μm, respectively, which represented a significant decrease as development progressed (*p < 0.05*) (Figure 1B). The average body widths observed in 1–5 days old specimens were 188.71, 187.85, 182.86, 171.55, and 164.86 μm, respectively which showed a significant reduction from day 3 to day 4 (*p < 0.05*) (Figure 1C). The average body widths of females showed a significant reduction in 3-, 4- and 5-day-old, while no significant difference in body lengths (*p* > *0.05*) (Figures 1B, C).

### Transcriptome sequencing result and data preprocessing

We obtained 357,765,304 bp raw data and 47,497,144 bp cleaned-up data. The alignment rate between reference genome and transcriptome assembly varied in the range of 77.20%–86.42% (Supplementary Material S7).

A total of 14,089 genes in the *S. chinensis* genome were annotated in the male transcriptome. Of these, 716 genes registered an expression level of 0 in all 15 male aphid samples (5 age groups of three replicates each). The data derived from the three replicates of 5-day-old samples, YX5.1, YX5.2 and YX5.3, were abnormal and could not be clustered with other samples. The cluster tree of the remaining 12 samples and the corresponding traits were showed in Figures 1D, E.

### Network topology analysis

**FIGURE 2:**
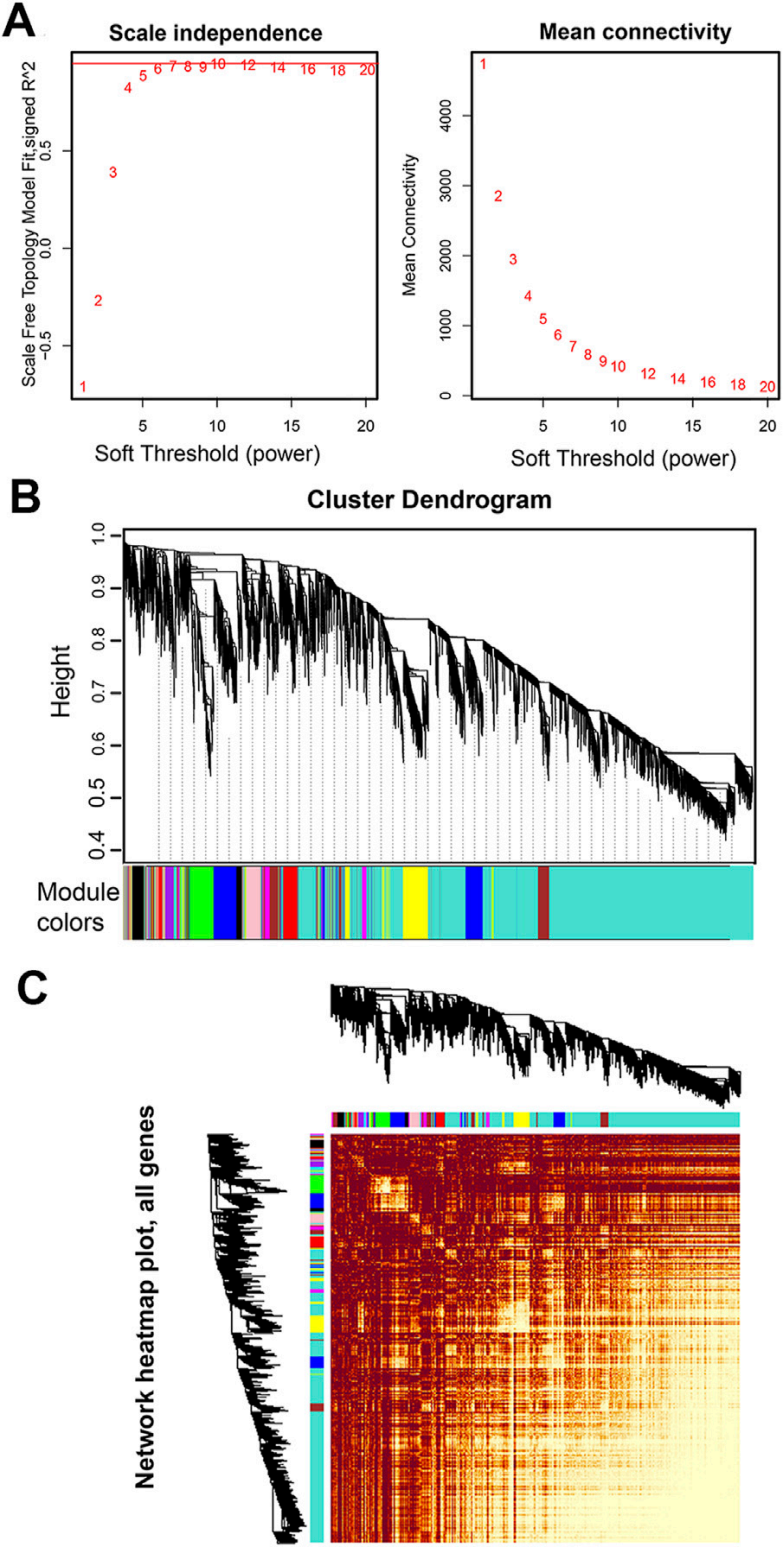
Network analysis of RNA-seq expression data in male *S. chinensis.* **(A)** Analysis of network topology for various soft-thresholding powers. Left panel: The scale free fit index (*y*-axis) as a function of the soft-thresholding power (*x*-axis); right panel: The mean connectivity (degree, *y*-axis) as a function of the soft-thresholding power (*x*-axis). The number crossed by the red horizontal line (‘7’ in the figure) is the lowest power that the scale-free topology fitting index curve flattens when it reaches a high value. **(B)** Dendrogram based on hierarchical clustering of genes, with dissimilarity based on the topological overlap, together with assigned module colors. **(C)** Network heatmap pot of all genes. The lighter the color, the stronger the correlation between genes.

An appropriate soft threshold power β value is essential for constructing a weighted gene network and to calculate adjacency by the co-expression similarity. Co-expression gene networks have the characteristics of scale-free networks. A scale free topology analysis of multiple β was performed using the “*pickSoftThreshold()*” function. The results showed that the lowest flattening of scale-free topological fitting exponential curve when it reaches a β high value of 7 (the scale free topological model is supposed to be 0.85) (Figure 2A). Therefore, we selected 7 as the β for subsequent network construction.

### Network construction and module detection

A co-expression network was constructed based on the optimal soft threshold of β = 7 to obtain a dissTOM of all genes (Figure 2C). The dissTOM algorithm generated a hierarchical clustering tree in which genes and gene modules were in the upper and lower parts, respectively (Figure 2B). The minimum gene number of each module was defined as 30, and 15 modules were divided according to the difference in the topological overlay, which were distinguished by different colors.

### Gene co-expression modules correlated with body size traits

**FIGURE 3:**
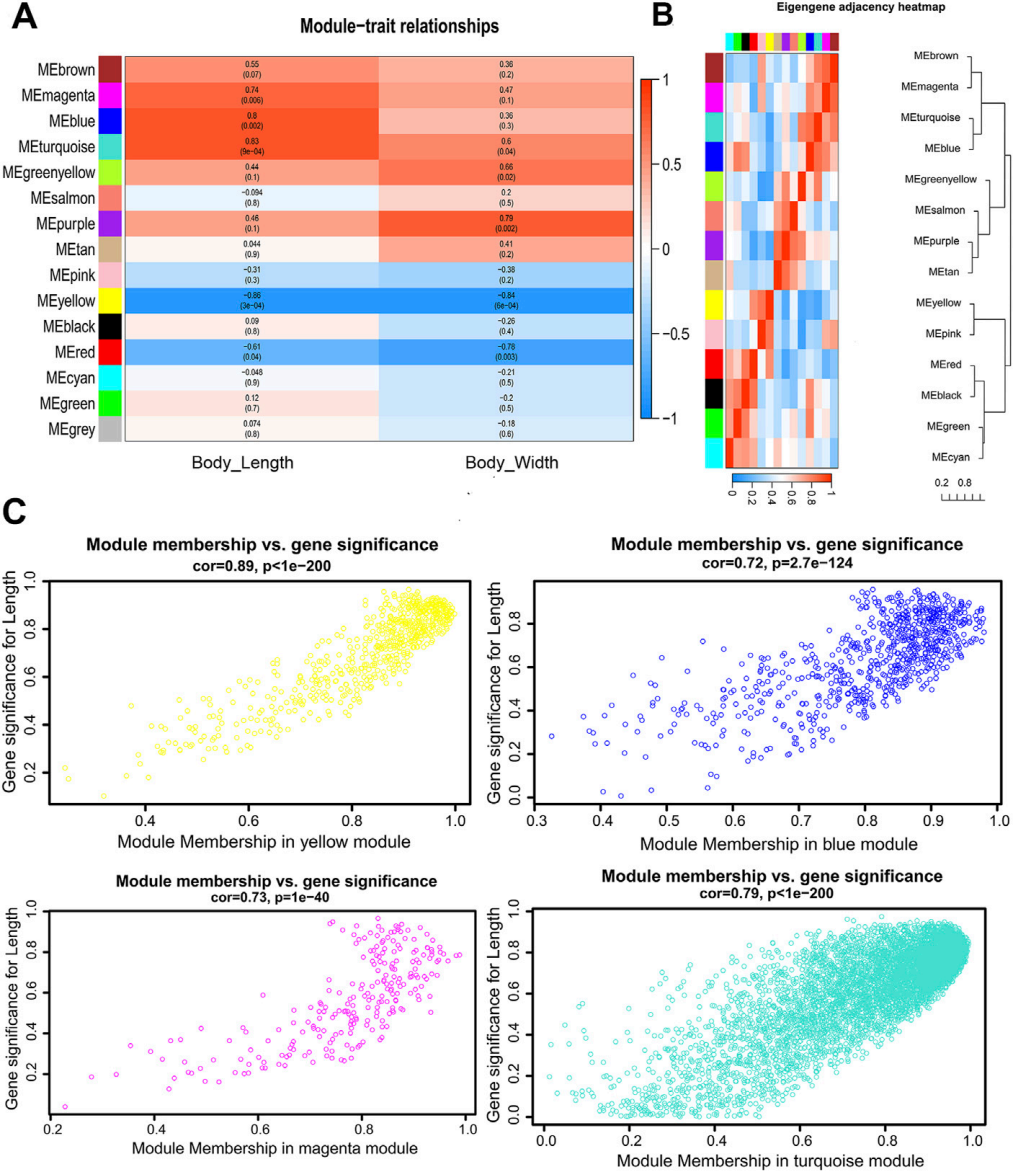
Visualization of the relationship between modules and body size traits of male *S. chinensis* **(A)** The relationship of module and trait. Each row corresponds to a module eigengene, each column corresponds to a trait, and each cell contains the corresponding correlation and *p*-value. **(B)** Hierarchical clustering dendrogram and the heatmap of the eigengenes. **(C)** Scatter plots of gene significance (GS) for male body length vs. module membership (MM) in the yellow, blue, magenta and turquoise modules.

Gene significance (GS) is defined as the correlation (absolute value) between genes and traits to quantify the contribution of a single gene to a trait (male body length). For each module, a quantitative measure of module membership (MM) is defined as the correlation of the module eigengene with gene expression characteristics. This allows us to quantify the degree of similarity for all genes of every module on the array. Gene modules that show high significance to male body length and module members with high correlation modules of interest were identified through calculating the GS and MM. The results were used to draw a color-coded table (Figure 3A) and a summary of network (Supplementary Material S5). Four modules with magenta, blue, turquoise and yellow coding have the highest association (*p* < 0.05) with male body length (Figure 3A). The hierarchical clustering tree of modules and the adjacency heatmap of each module revealed that the turquoise and blue modules have the highest correction (Figure 3B). The scatter plot of GS and MM showed a highly significant correlation between GS and MM in the four selected modules (Figure 3C).

### Module analysis using KEGG enrichment

**FIGURE 4:**
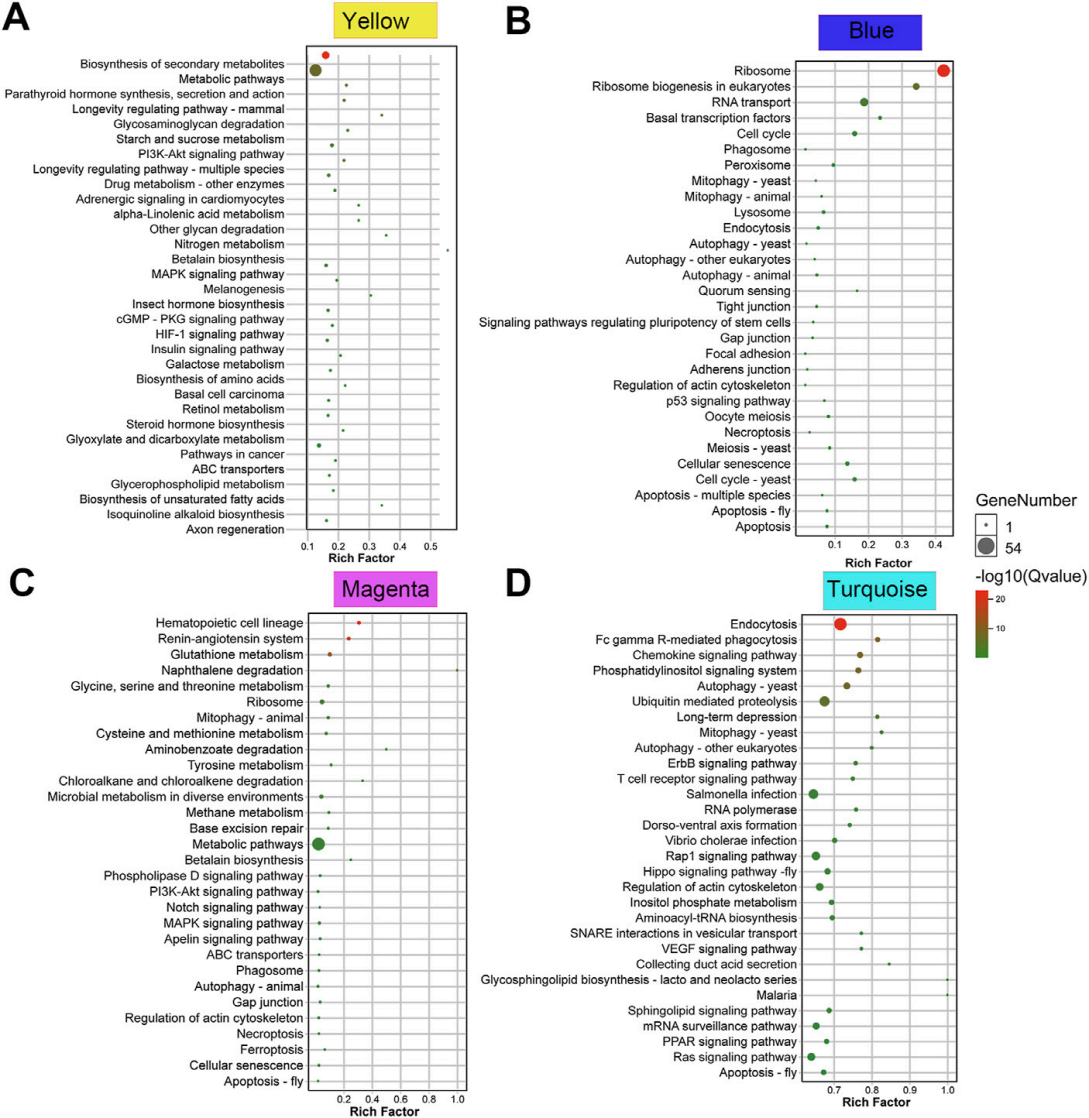
**(A–D)** KEGG enrichment analysis of magenta, blue, turquoise and yellow module of male *S. chinensis*.

To explore the biological functions of the modules related to male body length, the four selected modules, in magenta, blue, turquoise and yellow were analyzed using KEGG enrichment pathway. In the top 30 pathways, mitophagy, autophagy and apoptosis appeared in enrichment pathway of magenta, blue, and turquoise modules (Figures 4B–D). At the same time, a large number of pathways known to regulate body size were also found in the module enrichment data. For example, P13K-Akt, Notch and MAPK were presented in the magenta module (Figure 4C). P53 linked to the blue module. Hippo, PPAR and Ras were associated with the turquoise module (Figure 4D). P13K-Akt, Insulin and MAPK were a part of the yellow module (Figure 4A).

### Hub gene analysis

**FIGURE 5:**
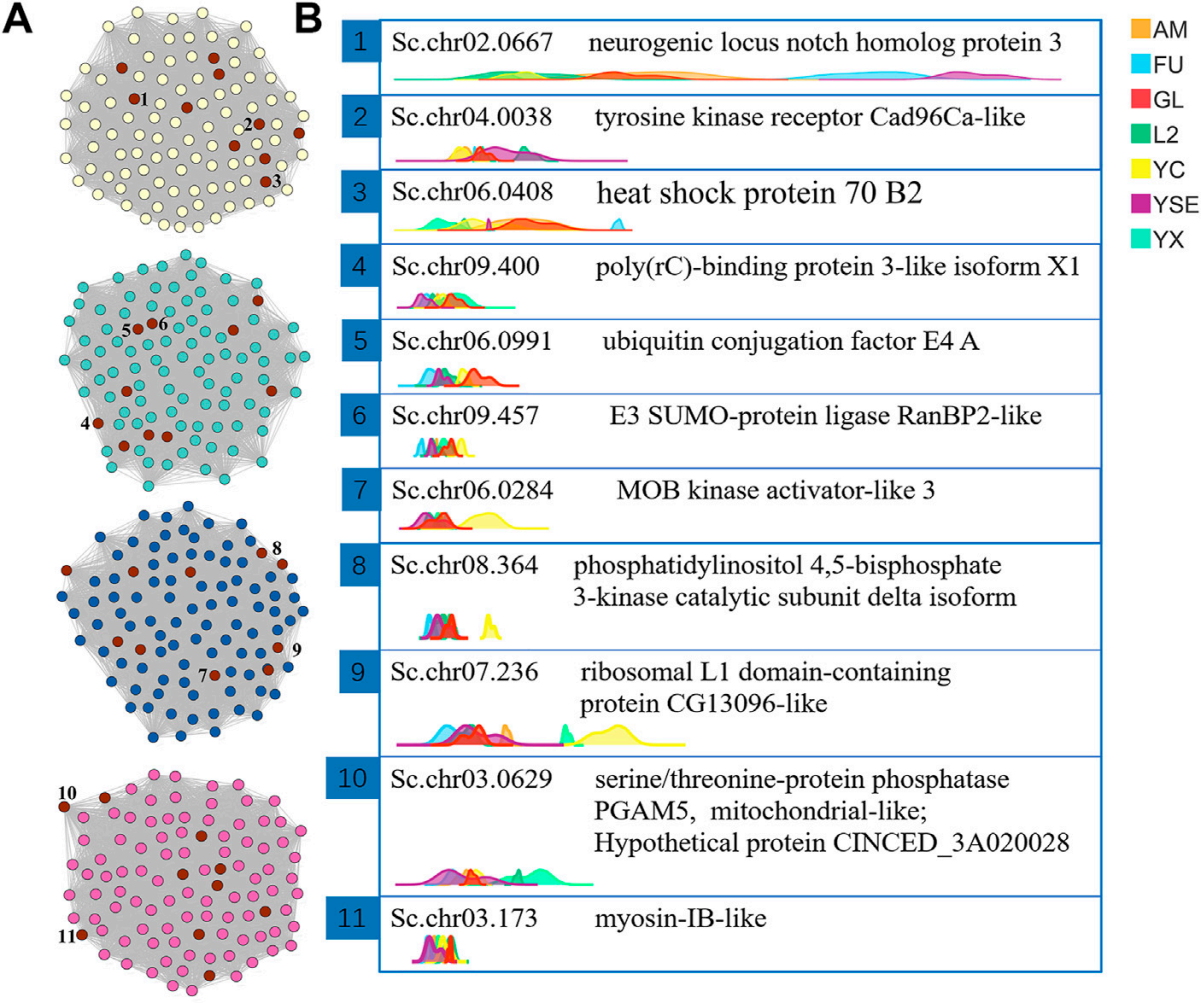
The visual network structure diagram and ridgeline plots of the screened hub genes related to body length of male *S. chinensis* **(A)** Visualization of network connections between the top 100 genes with the most connections in the yellow, turquoise, blue and magenta modules that were generated by the Cytoscape software. The nodes marked in red are the 10 most highly ranked nodes (genes) with the largest degree calculated by the CytoNAC plugin. The 1 to 10 numbers marked by the nodes are the selected hub genes. **(B)** Ridgeline plots of the expression levels of the selected hub genes in each sample showing gene ids and the protein functions annotated by NR. Abbreviations in the top right-hand corner represent different generations of *S. chinensis*. Abbreviations: AM, autumn migrant; FU, fundatrix; GL, fundatrigenia; L2, overwinter nymph; YC, female; YSE, spring migrant (sexuparae); YX, male.

Through the “*exportNetworkToCytoscape()*” function of WGCNA software, the topology network calculated earlier was exported as a network file that can be recognized by the Cytoscape software. In this study, the hub genes were screened based on the degree of each gene (called nodes in the network). The degree is a connection between one node and another. The larger the degree value of a node, the more connections the node has, which means that the gene connected with the most genes and was defined as a hub gene. The top 10 genes in the magenta, blue, turquoise and yellow modules were obtained using the CytoNAC weighted calculation, with a total of 40 genes (Figure 5A). Nr annotation was performed for 40 hub genes, and 11 of them that were predicted to act in autophagy and apoptosis were selected, including Sc.chr02.0667, Sc.chr04.0038, Sc.chr06.0408, Sc.chr09.400, Sc.chr06.0991, Sc.chr09.457, Sc.chr06.0284, Sc.chr08.364, Sc.chr07.236, Sc.chr03.0629, and Sc.chr03.173. From the ridgeline plots that show the expression levels of these 11 genes for each generation of *S. chinensis*, including autumn migrant, fundatrix, fundatrigenia, overwinter nymph, female, spring migrant (sexuparae) and male. We found eight of these genes had significant higher expression levels in males than those *S. chinensis* of other generations. These eight genes encode the heat shock protein 70 B2, poly(rC)-binding protein 3-like isoform X1, ubiquitin conjugation factor E4 A, E3 SUMO-protein ligase RanBP2-like, MOB kinase activator-like 3, phosphatidylinositol 4,5-bisphosphate 3-kinase catalytic subunit delta isoform, ribosomal L1 domain-containing protein CG13096-like and myosin-IB-like. Genes of Sc.chr09.457, Sc.chr06.0284, Sc.chr08.364, and Sc.chr07.236 in females also had higher expression levels than in other generations (Figure 5B).

## Discussion

The main purposes of WGCNA analysis of RNA sequence data are to identify gene modules and explore relationships between different modules and hub genes related to a specific trait (here: Male body length). In our study, four gene modules which appeared to be correlated with the body size changes in male of *S. chinensis* were identified using WGCNA analysis. Interestingly, most pathways identified in the four modules by KEGG analysis are associated with autophagy and apoptosis, suggesting that these pathways may be critical during this sexual phase of *S. chinensis* development. For example, IIS and TOR signals regulate autophagy in fat body of *Drosophila melanogaster* (Ryan et al., 2004). The PI3K signaling pathway is necessary for autophagy to promote survival during starvation in *Cryptococcus neoformans* (Hu et al., 2008). The mTOR kinase is a key element for autophagy induction. Pathways that activate mTOR, such as Akt, and MAPK signal pathways, inhibit autophagy. Pathways that inhibit mTOR, such as AMPK, and p53 signal pathways, promote autophagy (Neufeld, 2010; Alers et al., 2012; Russell et al., 2014). Thus, we conclude that the body size of male *S. chinensis* decreases after molting without feeding is probably related to autophagy and apoptosis.

Four topological centricities including degree centrality (DC), closeness centrality (CC), between centrality (BC) and eigenvector centrality (EC) are used to measure the closeness degree of nodes to the center of the network. In our study, the weighted DC index was used to sort the nodes in the network and screen for hub genes (Ding et al., 2022). Eleven of the identified hub genes were linked to autophagy and apoptosis. For example, the Sc.chr04.0038 gene encodes the tyrosine kinase receptor Cad96Ca, which probably functions in autophagy (Lampada et al., 2017). The Sc.chr02.0108 gene encodes an XK-related protein that has significant similarity to the ced-8 gene of *Caenorhabditis elegans* and this protein controls apoptosis timing (Stanfield and Horvitz, 2000). The Sc.chr06.0408 and Sc.chr04.0212 genes encode the heat shock proteins (HSP) 70 B2 and 75 KDa. These HSPs may directly affect apoptosis by intervening in the signal transduction pathway of apoptosis and they play an important role in the regulation of apoptosis (Ikwegbue et al., 2017). They also affect autophagy (Dokladny et al., 2015). Furthermore, we identified eight genes related to autophagy and apoptosis that have higher expression levels in males of the sexual generation compared to other asexual generations. In addition, we also found that these eight genes were highly expressed in females as well, especially Sc.chr09.457, Sc.chr06.0284, Sc.chr08.364, and Sc.chr07.236 (data not reported here). Female’s body size does not change significantly result from the volume of egg in female’s abdomen increased along with the increase of the age (Zhang and Tang, 1987). We speculate that it may have the same metabolism, autophagy and apoptosis processes as male, and the identified eight genes may play an important role for autophagy and apoptosis in both sexes.

Autophagy is a cellular response to starvation that generates autophagosomes to carry long-lived proteins and cellular organelles to lysosomes for degradation (Miklos, 2008). During autophagy, amino acids and other nutrients are recycled from long live proteins, organelles and other components in the cytoplasm to provide nutrients for necessary life activities (Tracy and Baehrecke, 2013). Autophagy is essential for balancing sources of energy at critical times in development and an effective response to nutrient stress and many other adverse stimulates in many animals including insects (Glick et al., 2010). Mobilization of stored nutrients from the larval fat body of *D. melanogaster*, can be induced by nutrient starvation (Ryan et al., 2004). When starvation stress occurs, the size of fat body cells of *D. melanogaster* larvae decreases by 90%, and at the same time, a large number of autophagy appears (Butterworth et al., 1965; Shi and Tong, 2022). Autophagy also occurred in the ovaries of *D. melanogaster* under nutritional stress and it is crucial for egg formation (Barth et al., 2011). Hibernation is a natural starvation period. No surprise, the autophagic activity of *Troglophilus neglectus* gradually increased in the early, middle and late overwintering (hibernation) stages (Lipovšek and Novak, 2016). Nutrient levels also modulate apoptosis in special cells, such as cartilage endplate stem cells, nucleus pulposus, gastric and colon cancer cells (Matthews et al., 2006; He et al., 2017; Liu et al., 2017). Both autophagy and apoptosis are parts of programmed cell death, while autophagy can control apoptosis by increasing or decreasing the possibility of apoptosis. Conversely, apoptotic processes can increase or decrease autophagy as well (Jacob and Andrew, 2011). Autophagy and apoptosis may inhibit each other through multiple pathways or can be independently regulated by co-occurring signals. For *S. chinensis*, both male and female of the only sexual generation in its life cycle have no functional mouthparts and cannot ingest food. It makes sense that genes associated with autophagy and apoptosis were identified in the current study with *S. chinensis*, especially in males in which body size reduction was observed as they age. In order to achieve sexual maturity and mating without feeding, male aphids (and females as well) may utilize autophagy and apoptosis to provide energy and components for developmental processes.

## Data Availability

The datasets presented in this study can be found in online repositories. The names of the repository/repositories and accession number(s) can be found in the article/Supplementary Material
