# Supplementary material for: Candidate genes potentially involved in molting and body size reduction in the male of the horned gall aphid, Schlechtendalia chinensis
Source: Front Physiol. 2023 Feb 6;14:1097317. doi: 10.3389/fphys.2023.1097317 (PMC9940790; doi:10.3389/fphys.2023.1097317)
Supplement: Supplementary file 1 [file DataSheet4.docx]

# References:

# [1] Langfelder, P.; Horvath, S. WGCNA: an R Package for Weighted Correlation

# Network Analysis. BMC Bioinformatics 2008, 9, 559, doi:https://doi.org/10.1186/1471-2105-9-559.

# [2] Langfelder, P.; Horvath, S. Tutorials for the WGCNA Package.

# Available online: https://horvath.genetics.ucla.edu/html/CoexpressionNetwork/Rpackages/WGCNA/Tutorials/index.html (accessed on June 15).

#

# If necessary, change the path below to the directory where the data files are stored.

# "." means current directory. On Windows use a forward slash / instead of the usual \.

workingDir = "."

setwd(workingDir)

# Load the WGCNA package

library(dynamicTreeCut)

library(fastcluster)

library(WGCNA)

library(stringr)

library(reshape2)

# The following setting is important, do not omit.

options(stringsAsFactors = FALSE)

# Allow multi-threading within WGCNA.

enableWGCNAThreads()

# Read in the FPKM value list of all samples data set

YXData = read.csv("YXS1.csv")

# Take a quick look at what is in the data set:

dim(YXData)

names(YXData)

# We now remove the auxiliary data and transpose the expression data for further analysis.

datExpr0 = as.data.frame(t(YXData[, -c(1:1)]))

names(datExpr0) = YXData$geneid

rownames(datExpr0) = names(YXData)[-c(1:1)]

# We first check for genes and samples with too many missing values:

gsg = goodSamplesGenes(datExpr0, verbose = 3)

gsg$allOK

# If the last statement returns TRUE, all genes have passed the cuts.

# If not, we remove the offending genes and samples from the data:

if (!gsg$allOK)

{

# Optionally, print the gene and sample names that were removed:

if (sum(!gsg$goodGenes)>0)

printFlush(paste("Removing genes:", paste(names(datExpr0)[!gsg$goodGenes], collapse = ",")));

if (sum(!gsg$goodSamples)>0)

printFlush(paste("Removing samples:", paste(rownames(datExpr0)[!gsg$goodSamples], collapse = ",")));

# Remove the offending genes and samples from the data:

datExpr0 = datExpr0[gsg$goodSamples, gsg$goodGenes]

}

# Next we cluster the samples (in contrast to clustering genes that will come later)

# to see if there are any obvious outliers.

sampleTree = hclust(dist(datExpr0), method = "average")

# Plot the sample tree: Open a graphic output window of size 12 by 9 inches

# The user should change the dimensions if the window is too large or too small.

sizeGrWindow(12,9)

par(cex = 0.6)

par(mar = c(0,4,2,0))

plot(sampleTree, main = "Sample clustering to detect outliers", sub="", xlab="", cex.lab = 2, cex.axis = 2, cex.main = 2, cex=2, lwd=2)

# It appears there is one outlier (sample EA1). Choose a height cut that will remove the offending sample,

# say 80000 (the red line in the plot), and use a branch cut at that height.

# Plot a line to show the cut

abline(h = 25000, col = "red",lwd=2)

clust = cutreeStatic(sampleTree, cutHeight = 25000, minSize = 10)

table(clust)

# clust 1 contains the samples we want to keep.

keepSamples = (clust==1)

datExpr = datExpr0[keepSamples, ]

nGenes = ncol(datExpr)

nSamples = nrow(datExpr)

# We now read in the trait data and match the samples for which they were measured to the expression samples.

traitData = read.csv("YXS2.csv")

dim(traitData)

names(traitData)

# remove columns that hold information we do not need.

allTraits = traitData[, -c(1)]

allTraits = allTraits[, c(1: 3) ]

dim(allTraits)

names(allTraits)

# Form a data frame analogous to expression data that will hold the traits.

lengthSamples = rownames(datExpr)

traitRows = match(lengthSamples, allTraits$Sample_Name)

datTraits = allTraits[traitRows, -1]

rownames(datTraits) = allTraits[traitRows, 1]

collectGarbage()

# Before we continue with network construction and module detection,

# we visualize how the traits relate to the sample dendrogram.

# Re-cluster samples

sampleTree2 = hclust(dist(datExpr), method = "average")

# Convert traits to a color representation: white means low, red means high, grey means missing entry

traitColors = numbers2colors(datTraits, signed = FALSE)

# Plot the sample dendrogram and the colors underneath.

plotDendroAndColors(sampleTree2, traitColors, groupLabels = names(datTraits), main = "Sample dendrogram and trait heatmap")

# Calculate the median absolute deviation (MAD) for each gene and sort by this value.

# Retain genes with the top 75% of MAD that are greater than 0.01.

m.mad<-apply(datExpr,2,mad)

m.mad

dataExprMad<-datExpr[,which(m.mad>max(quantile(m.mad,probs=seq(0,1,0.25))[2],0.01))]

# Choose a set of soft-thresholding powers

powers = c(c(1:10), seq(from = 12, to=20, by=2))

# Call the network topology analysis function

sft = pickSoftThreshold(dataExprMad, powerVector = powers, verbose = 5)

# Plot the results:

sizeGrWindow(9, 5)

par(mfrow = c(1,2))

cex1 = 0.9

# Scale-free topology fit index as a function of the soft-thresholding power

plot(sft$fitIndices[,1], -sign(sft$fitIndices[,3])*sft$fitIndices[,2], xlab="Soft Threshold (power)",ylab="Scale Free Topology Model Fit,signed R^2",type="n", main = paste("Scale independence"))

text(sft$fitIndices[,1], -sign(sft$fitIndices[,3])*sft$fitIndices[,2], labels=powers,cex=cex1,col="red")

# this line corresponds to using an R^2 cut-off of h

abline(h=0.85,col="red")

# Mean connectivity as a function of the soft-thresholding power

plot(sft$fitIndices[,1], sft$fitIndices[,5], xlab="Soft Threshold (power)",ylab="Mean Connectivity", type="n", main = paste("Mean connectivity"))

text(sft$fitIndices[,1], sft$fitIndices[,5], labels=powers, cex=cex1,col="red")

# Block-wise network construction and module detection

net = blockwiseModules(dataExprMad, maxBlockSize = 100000, power = 7, TOMType = "unsigned", minModuleSize = 30, reassignThreshold = 0, mergeCutHeight = 0.25, numericLabels = TRUE, pamRespectsDendro = FALSE, saveTOMs = TRUE, saveTOMFileBase = "LengthTOM", verbose = 3)

# To see how many modules were identified and what the module sizes are:

table(net$colors)

# The dendrograms can be displayed together with the color assignment using the following code:

# open a graphics window

sizeGrWindow(12, 9)

mergedColors = labels2colors(net$colors)

plotDendroAndColors(net$dendrograms[[1]], mergedColors[net$blockGenes[[1]]], "Module colors", dendroLabels = FALSE, hang = 0.03, addGuide = TRUE, guideHang = 0.05)

moduleLabels = net$colors

moduleColors = labels2colors(net$colors)

MEs = net$MEs

geneTree = net$dendrograms[[1]]

# Relating modules to external traits

# Define numbers of genes and samples

nGenes = ncol(dataExprMad)

nSamples = nrow(dataExprMad)

# Recalculate MEs with color labels

MEs0 = moduleEigengenes(dataExprMad, moduleColors)$eigengenes

MEs = orderMEs(MEs0)

moduleTraitCor = cor(MEs, datTraits, use = "p")

moduleTraitPvalue = corPvalueStudent(moduleTraitCor, nSamples)

# We color code each association by the correlation value:

sizeGrWindow(10,6)

# Will display correlations and their p-values

textMatrix = paste(signif(moduleTraitCor, 2), "\n(", signif(moduleTraitPvalue, 1), ")", sep = "")

dim(textMatrix) = dim(moduleTraitCor)

par(mar = c(6, 8.5, 3, 3))

# Display the correlation values within a heatmap plot

labeledHeatmap(Matrix = moduleTraitCor, xLabels = names(datTraits), yLabels = names(MEs), ySymbols = names(MEs), colorLabels = FALSE, colors = blueWhiteRed(50), textMatrix = textMatrix, setStdMargins = FALSE, cex.text = 0.5, zlim = c(-1,1), main = paste("Module-trait relationships"))

# Define variable SecretWax containing the Secret_wax column of datTrait

Length = as.data.frame(datTraits$Body_Length)

names(Length)="Length"

# names (colors) of the modules

modNames = substring(names(MEs), 3)

geneModuleMembership = as.data.frame(cor(dataExprMad, MEs, use = "p"))

MMPvalue = as.data.frame(corPvalueStudent(as.matrix(geneModuleMembership), nSamples))

names(geneModuleMembership) = paste("MM", modNames, sep="")

names(MMPvalue) = paste("p.MM", modNames, sep="")

LengthSignificance = as.data.frame(cor(dataExprMad, Length, use = "p"))

GSPvalue1 = as.data.frame(corPvalueStudent(as.matrix(LengthSignificance), nSamples))

names(LengthSignificance) = paste("GS.", names(Length), sep="")

names(GSPvalue1) = paste("p.GS.", names(Length), sep="")

# We plot a scatterplot of Gene Significance vs. Module Membership in the blue module:

module = "blue"

column = match(module, modNames)

moduleGenes = moduleColors==module

sizeGrWindow(7, 7)

par(mfrow = c(1,1))

verboseScatterplot(abs(geneModuleMembership[moduleGenes, column]), abs(LengthSignificance[moduleGenes, 1]), xlab = paste("Module Membership in", module, "module"), ylab = "Gene significance for Length", main = paste("Module membership vs. gene significance\n"), cex.main = 1.2, cex.lab = 1.2, cex.axis = 1.2, col = module)

# The plots of other modules are generated similarly to the above methods

# Summary output of network analysis results

annot = read.csv(file = "YXS3.csv")

dim(annot)

names(annot)

probes = names(dataExprMad)

probes2annot = match(probes, annot$Genes)

# The following is the number or probes without annotation:

sum(is.na(probes2annot))

# Should return 0.

# Create the starting data frame

geneInfo0 = data.frame(geneid = probes, Nr = annot$Nr[probes2annot], KEGG = annot$KEGG[probes2annot], moduleColor = moduleColors, LengthSignificance, GSPvalue1)

# Order modules by their significance for SecretWax

modOrder = order(-abs(cor(MEs, Length, use = "p")))

# Add module membership information in the chosen order

for (mod in 1:ncol(geneModuleMembership))

{

oldNames = names(geneInfo0)

geneInfo0 = data.frame(geneInfo0, geneModuleMembership[, modOrder[mod]], MMPvalue[, modOrder[mod]]);

names(geneInfo0) = c(oldNames, paste("MM.", modNames[modOrder[mod]], sep=""), paste("p.MM.", modNames[modOrder[mod]], sep=""))

}

load("~/WWW/01/GelTOM-block.1.RData")

TOM <- as.matrix(TOM)

dissTOM <-1-TOM

plotTOM = dissTOM^7

diag(plotTOM) = NA

sizeGrWindow(9,9)

TOMplot(plotTOM, geneTree, moduleColors, main = "Network heatmap plot, all genes")

# Order the genes in the geneInfo variable first by module color, then by geneTraitSignificance

geneOrder = order(geneInfo0$moduleColor, -abs(geneInfo0$GS.Length))

geneInfo = geneInfo0[geneOrder, ]

write.csv(geneInfo, file = "YXS5.csv")

MET = orderMEs(cbind(MEs, Length))

MEs = net$MEs

MEs_col = MEs

colnames(MEs_col) = paste0("ME", labels2colors(as.numeric(str_replace_all(colnames(MEs),"ME",""))))

MEs_col = orderMEs(MEs_col)

# Plot the dendrogram

plotEigengeneNetworks(MEs_col, "Eigengene dendrogram", marDendro = c(0,4,2,0), plotHeatmaps = FALSE)

# Plot the heatmap matrix (note: this plot will overwrite the dendrogram plot)

plotEigengeneNetworks(MEs_col, "Eigengene adjacency heatmap", marDendro = c(3,3,2,4),marHeatmap = c(3,4,2,2), plotDendrograms = T, xLabelsAngle = 90)

# Exporting to Cytoscape

lnames = load(file = "LengthTOM-block.1.RData")

TOM <- as.matrix(TOM)

probes = colnames(dataExprMad)

dimnames(TOM) <- list(probes, probes)

load(file = "LengthTOM-block.1.RData")

TOM <- as.matrix(TOM)

dissTOM <-1-TOM

plotTOM = dissTOM^7

diag(plotTOM) = NA

sizeGrWindow(9,9)

TOMplot(plotTOM, geneTree, moduleColors, main = "Network heatmap plot, all genes")

# Read in the annotation file

annot = read.csv(file = "YXS3.csv")

# Select modules

modules = c("yellow")

inModule = is.finite(match(moduleColors, modules))

modProbes = probes[inModule]

modGenes = annot$Nr[match(modProbes, annot$geneid)]

# Select the corresponding Topological Overlap

modTOM = TOM[inModule, inModule]

dimnames(modTOM) = list(modProbes, modProbes)

# Export the network into edge and node list files Cytoscape can read

cyt = exportNetworkToCytoscape(modTOM,

edgeFile = paste("CytoscapeInput-yellow-edges-", paste(modules, collapse="-"), ".txt", sep=""),

nodeFile = paste("CytoscapeInput-yellow-nodes-", paste(modules, collapse="-"), ".txt", sep=""),

weighted = TRUE, threshold = 0.02, nodeNames = modProbes, altNodeNames = modGenes,

nodeAttr = moduleColors[inModule])

# The operation of other modules exported as Cytoscape files is similar.

# From the Cytoscape node file just generated, delete the line with the node gene annotated as NA:

CytoscapeInput.yellow.edges.yellow <- read.delim("CytoscapeInput-yellow-edges-yellow.txt")

CytoscapeInput.yellow.edges.yellow <-CytoscapeInput.yellow.edges.yellow[complete.cases(CytoscapeInput.yellow.edges.yellow[,5:6]),]

dim(CytoscapeInput.yellow.edges.yellow)

write.csv(CytoscapeInput.yellow.edges.yellow,file="CytoscapeInput-yellow-edges-yellow-rmNA.txt",quote=F)

# The operation method of other module node files is similar.

# Calculate the degree of each node, that is, how many edges each node has:

Cyellowdegree<-data.frame(table(CytoscapeInput.yellow.edges.yellow$fromNode))

write.csv(Cyellowdegree,file="Cyellow-degree.txt")

# Use Excel to sort the degrees from largest to smallest

# Use Cytoscape to select the top 100 nodes with the highest degree for subsequent screening
